# Evidence of the Involvement of a Cyclase Gene in the Biosynthesis of Ochratoxin A in *Aspergillus carbonarius*

**DOI:** 10.3390/toxins13120892

**Published:** 2021-12-13

**Authors:** Massimo Ferrara, Antonia Gallo, Carla Cervini, Lucia Gambacorta, Michele Solfrizzo, Scott E. Baker, Giancarlo Perrone

**Affiliations:** 1 Institute of Sciences of Food Production (ISPA), National Research Council (CNR), 70126 Bari, Italy; lucia.gambacorta@ispa.cnr.it (L.G.); michele.solfrizzo@ispa.cnr.it (M.S.); giancarlo.perrone@ispa.cnr.it (G.P.); 2 Institute of Sciences of Food Production (ISPA), National Research Council (CNR), 73100 Lecce, Italy; antonia.gallo@ispa.cnr.it; 3 Applied Mycology Group, Environment and AgriFood Theme, Cranfield University, Cranfield MK43 0AL, UK; carla.cervini@cranfield.ac.uk; 4 Functional and Systems Biology Group, Environmental Molecular Sciences Division, Pacific Northwest National Laboratory, Richland, WA 99354, USA; scott.baker@pnnl.gov; 5 DOE Joint Bioenergy Institute, Emeryville, CA 94608, USA

**Keywords:** OTA, biosynthetic cluster, SnoaL domain, polyketide cyclases, CRISPR/Cas9

## Abstract

Ochratoxin A (OTA) is a well-known mycotoxin with wide distribution in food and feed. Fungal genome sequencing has great utility for identifying secondary metabolites gene clusters for known and novel compounds. A comparative analysis of the OTA-biosynthetic cluster in *A. steynii, A. westerdijkiae, A. niger, A. carbonarius*, and *P. nordicum* has revealed a high synteny in OTA cluster organization in five structural genes (*otaA*, *otaB,* *ota*, *otaR1*, and *otaD*). Moreover, a recent detailed comparative genome analysis of Aspergilli OTA producers led to the identification of a cyclase gene, *otaY*, located in the OTA cluster between the *otaA* and *otaB* genes, encoding for a predicted protein with high similarity to SnoaLs domain. These proteins have been shown to catalyze ring closure steps in the biosynthesis of polyketide antibiotics produced in *Streptomyces*. In the present study, we demonstrated an upregulation of the cyclase gene in *A. carbonarius* under OTA permissive conditions, consistent with the expression trends of the other OTA cluster genes and their role in OTA biosynthesis by complete gene deletion. Our results pointed out the involvement of a cyclase gene in OTA biosynthetic pathway for the first time. They represent a step forward in the understanding of the molecular basis of OTA biosynthesis in *A. carbonarius*.

## 1. Introduction

Ochratoxin A (OTA) is a well-known mycotoxin with wide distribution in food and feed, including cereal products, grapes and by-products, coffee, beverages, cocoa, nuts, dried fruits, and cured meat [1,2]. Ochratoxin A is a secondary fungal metabolite with several toxicological effects (such as hepatotoxic, nephrotoxic, teratogenic, embryotoxic, etc.) [3,4,5]. Ochratoxin A is produced by many species of the genus *Aspergillus* and *Penicillium* [6]. One of the main OTA producing species is *Aspergillus carbonarius*, known not only for its high capacity for producing OTA and its high percentage of toxigenic strains but also because it is considered the main species for OTA contamination of grapes in the vineyard worldwide [7].

Fungal genome sequencing has great utility for the identification of secondary metabolite gene clusters for known and novel compounds [8,9]. This kind of genomic approach has led to the identification of the clustered OTA biosynthetic genes, primarily in *A. niger*, and subsequently in *A. carbonarius* [10,11] and recently in several other OTA producing species [12,13]. In particular, the genomic analysis of *A. carbonarius* revealed the crucial role of three genes (*otaA*-AcOTApks, *otaB*-AcOTAnrps, and *otaD*-AcOTAhal) in the OTA biosynthesis [14,15,16]. Further, the involvement of a P450 cytochrome oxidase (*otaC*) and a bZIP transcription factor (*otaR1*) has been evidenced in six different OTA-producing species [12,17]. The essential role of these five core genes in OTA biosynthesis has also been consolidated by the gene knockout approach. Moreover, a comparative analysis of OTA-biosynthetic clusters in *A. steynii*, *A. westerdijkiae*, *A. niger*, *A. carbonarius*, and *P. nordicum* has revealed a high synteny in cluster organization [13]. A detailed comparative analysis of Aspergilli genomes has recently led us to identify a gene whose sequence encoded a predicted protein similar to bacterial polyketide cyclases. This gene is located in the OTA cluster between the *otaA* and *otaB* genes; it is present in the genome sequences of all currently sequenced OTA-producing fungi, and it was hypothesized to be involved in OTA production [12]. In particular, this gene encodes a protein with high similarity to SnoaLs domain. Largely characterized in bacteria, SnoaL domain-containing proteins have been characterized as small polyketide cyclases composed of about 140 amino acids. These proteins have been shown in *Streptomyces* to catalyze cyclization steps in the biosynthesis of polyketide antibiotics [18]. Concerning the OTA biosynthetic pathway, the cyclization process that leads to the formation of the heterocyclic structure of OTβ, in the initial biosynthetic steps, has remained unclear. Our identification of this SnoaL like protein—named *otaY*—along with the gene expression analysis suggested its putative involvement in the ring closure step of the OTA polyketide backbone [12]. Based on these results, we confirmed the role of *otaY* gene in OTA biosynthesis by gene disruption. In order to do this, we used a CRISPR/Cas9 mutagenesis approach to disrupt the cyclase gene in the strain of *A. carbonarius* ITEM 5010.

CRISPR (Clustered Regularly Interspaced Short Palindromic Repeats)–Cas9 (CRISPR-associated protein 9) is used by bacteria and archaea as a defense against viral infection and has grown into a revolutionary genome editing tool, which induces targeted DNA double strand breaks (DSBs). This gene editing system consists of two components: the Cas9 nuclease and a single guide RNA (sgRNA) that drives the Cas9/RNA complex to a specific target. Once the DNA double-strand is cleaved at the target site by Cas9 nuclease activity, both ends can subsequently be repaired by the non-homologous end joining (NHEJ) or homology directed repair (HDR) pathway. Due to the simplicity, efficiency, and the possibility of introducing mutations in multiple genes in one step, CRISPR/Cas9 has become a fast-growing genome editing tool in fungi [19]. It has been developed into a powerful technology that has been applied to various filamentous fungi, including *A. nidulans* and *A. aculeatus* [20], *A. oryzae* [21], *A. fumigatus* [22,23], *A. niger* [24], *A. carbonarius* [25], *Neurospora crassa* [26], *Alternaria alternata* [27], *Fusarium oxysporum* [28], and *F. graminearum* [29]. One of the recent advances in this technique is the direct delivery of Cas9/RNA complex into the cell [30,31,32,33,34]. Therefore, the need to express the Cas9 protein in the fungal host cell and assemble specific expression cassettes for each sgRNA is avoided by the direct transformation of fungal protoplasts with in vitro-assembled ribonucleoproteins (RNPs). The CRISPR/Cas9 system is particularly effective for complete gene deletions when coupled with dual in vitro-assembled Cas9–RNPs that target the upstream and downstream regions of a gene.

This technique offers the advantage of minimizing the risk of modifying adjacent gene sequences and targeting clustered genes located very close to each other, as in the genomic region hosting secondary metabolite clusters.

In the present study, we demonstrate the role of the *otaY* gene by complete gene deletion using the CRISPR/Cas9 approach. Moreover, the expression level of the cyclase gene is investigated to demonstrate its correlation to the kinetics of OTA accumulation and the expression profile of the other OTA biosynthetic genes. Our results point out for the first time the involvement of a new gene in the biosynthetic pathway of OTA in *A. carbonarius* and represent a knowledge advancement in the molecular basis of OTA biosynthesis.

## 2. Results

### 2.1. Gene Expression Analysis

**Figure 1:**
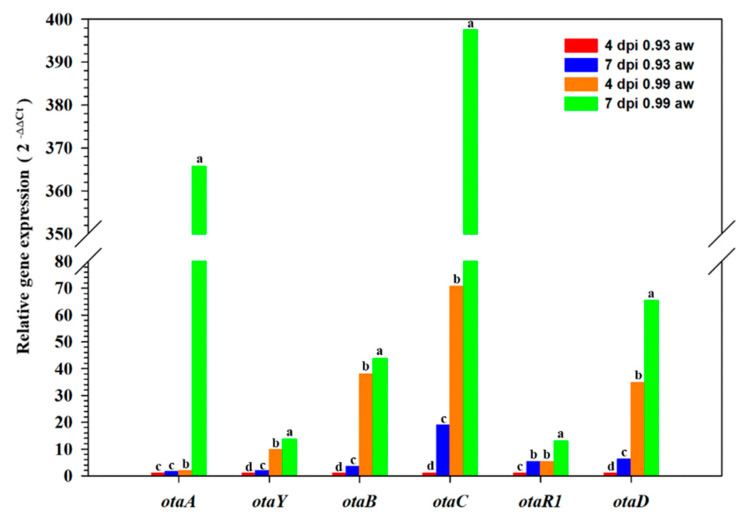
qRT-PCR analysis of OTA related genes in *A. carbonarius* ITEM 5010 at 18–31 °C. The reported gene expression under OTA permissive (0.99 aw) and not permissive (0.93 aw) was related to OTA not permissive (0.93 aw) conditions at 4-days post-inoculation (dpi). Relative gene expression analysis was performed in triplicates. Letters indicate significantly different means according to Tukey’s test (*p* ≤ 0.05).

While expression of *otaY* was confirmed previously from various OTA-producing fungi [12], we explored the expression of this cyclase-encoding gene relative to the other OTA biosynthetic cluster genes at multiple time points in *A. carbonarius* ITEM 5010. The qRT-PCR analysis of *otaY* gene and other OTA cluster genes (*otaA*, *otaB*, *otaC*, *otaR1*, and *otaD*) demonstrated an upregulation of the cyclase gene in *A. carbonarius* ITEM 5010 under OTA permissive conditions, in terms of growth conditions (medium and temperature). Furthermore, the modulation of the cyclase-encoding gene expression levels was consistent with the expression trends of the other OTA cluster genes at 4 and 7 dpi (Figure 1).

### 2.2. Selection and Analysis of ∆otaY Mutants

**Figure 2:**
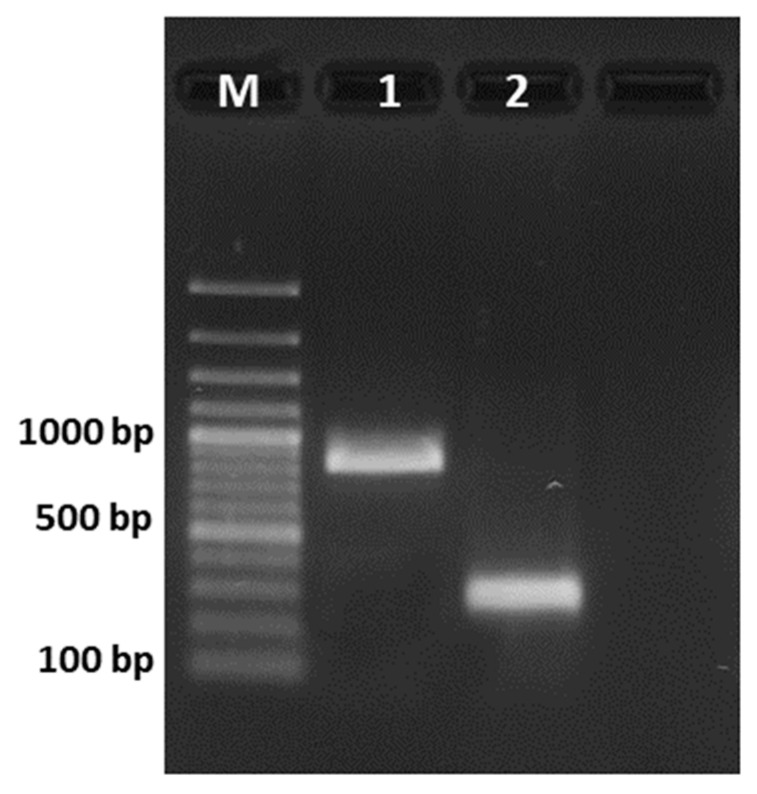
PCR amplification from genomic DNA of deletion site from wild-type ITEM 5010 (1) and AC2021 ∆*otaY* mutant strain (2). M = 100 bp ladder.

To delete the coding sequence of the cyclase gene *otaY*, we designed two sgRNAs (Table 1) that directed Cas9 RNPs complex to the 5’ and 3’-UTR regions of the *otaY* coding sequence. Based on predicted PAM sites and sgRNA scores, two sgRNAs were selected: the sgRNAotaY_up, located 172 bp upstream of the *otaY* start codon, and the sgRNAotaY_dw, located 32 bp downstream of the stop codon. After protoplast transformation, eight putative ∆*otaY* transformants were isolated on hygB medium. The putative ∆*otaY* mutants were PCR screened by amplification of the genomic sequence surrounding the excision sites. The sequencing of amplified regions obtained from the wild-type strain and ∆*otaY* mutant strains, represented by the selected strain AC2021 in Figure 2, resulted in amplicons of 916 bp and 292 bp, respectively, revealing the complete deletion of *otaY* gene and the lack of integration of the *hygB* resistance cassette in the *otaY* deletion site. Nevertheless, all the analyzed mutants were able to grow on *hygB* selective media and did not produce OTA (data not shown). In addition, sequence analysis of the amplified regions did not evidence any sequence alteration of UTRs regulatory regions of the genes adjacent to gene *otaY* (*otaA* and *otaB*). No significant phenotypic differences in terms of sporulation rate, pigmentation, and growth rate were observed among the wild-type and mutant strains grown on Czapek yeast extract agar (CYA), malt extract agar (MEA), and yeast extract sucrose (YES) agar at 25 °C in the dark after three and five days of incubation (Figure S1).

### 2.3. Genome Sequencing

The genome of the selected AC2021 Δ*otaY* mutant strain was sequenced and the de-novo assembly of quality filtered reads obtained from the sequencing generated 1461 contigs. The full sequencing dataset is available at Bioproject PRJNA738506. The analysis enabled the exact mapping of the excision site of the two RNP complexes. In detail, the comparative analysis with the sequence genome of *A. carbonarius* ITEM 5010 (Aspca3- https://mycocosm.jgi.doe.gov/Aspca3/Asp-ca3.home.html, accessed on 17 November 2021) revealed the complete deletion of *otaY* gene occurring between the position 951,737 and 952,362 of the original scaffold_12 of the wild-type strain, resulting in a deletion of 624 bp in the mutant strain. The deleted chromosomal segment included the full length *otaY* gene and was congruent with PCR analysis and Sanger sequencing of the region spanning the predicted deletion sites as reported in Figure 2. Analysis of UTRs regions of *otaA* and *otaB* gene surrounding the cyclase gene as well as sequence analysis of the entire genomic region of OTA cluster did not reveal any other nucleotide modification in the Δ*otaY* mutant strain. With regard to the *hygB* marker gene, its integration occurred between the positions 1,805,697 and 1,805,702 of the original scaffold_7 of the wild-type strain 5010. Upstream of the integration site is a gene encoding a peroxisomal NUDIX hydrolase (protein ID 396812) and downstream is a gene encoding a DNA binding protein (protein ID 207537). The integration of the *hygB* gene did not alter the coding sequences of genes surrounding the *hygB* integration site.

### 2.4. Expression Analysis of OTA Cluster Genes in ∆otaY Mutant Strain

The RT-PCR analysis of transcription of OTA cluster genes in strains ITEM 5010 and AC2021 after 4 dpi on MM agar medium (see oligonucleotides used in Table 2) revealed the expression of the complete set of genes in wild-type strain ITEM 5010 and the absence of the *otaY* transcript only in the mutant strain AC2021 (data not shown).

**Figure 3:**
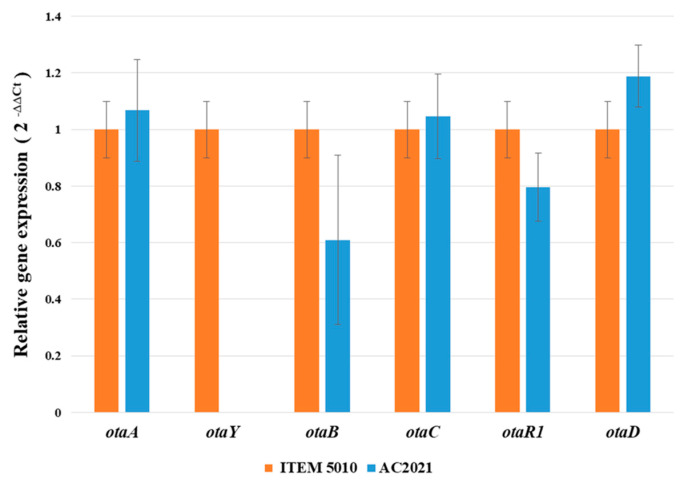
qRT-PCR analysis of OTA related genes in the wild-type ITEM 5010 and AC2021 ∆*otaY* mutant strain of *A. carbonarius*. Gene expressions were evaluated on MM agar plates after 4 days of incubation at 25 °C in the dark. The reported relative gene expression (2^−∆∆Ct^) was related to expression levels of each gene in wild-type ITEM 5010. Relative gene expression analysis was performed in triplicates. Bars indicate standard error.

Moreover, qRT-PCR analysis of *otaA, otaB, otaC, otaR1*, and *otaD* genes confirmed that the deletion of *otaY* gene did not prevent or significantly alter the expression of the other OTA cluster biosynthetic genes (Figure 3).

### 2.5. OTA Analysis

**Figure 4:**
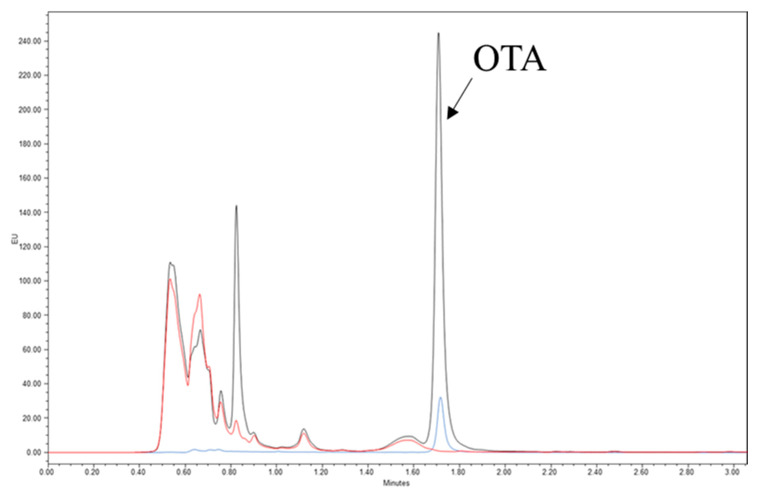
UPLC-FLD chromatograms of OTA standard solution (1018 ng/g) (blue trace), extract of *A. carbonarius* wild-type strain ITEM 5010 (6467 ng/g) (black trace), and extract of *A. carbonarius* ∆*otaY* deletion mutant strain AC2021 (red trace). Retention times = OTA 1.72 min.

The production of OTA by UPLC-FLD in mycelium collected after growth on 0.99 and 0.93 aw Grape Juice Medium (GJM) at 18/31 °C under 10 h/14 h dark/light photoperiod accounted for the OTA permissive condition (0.99 aw GJM) in 472.23 and 452.82 ng/g at 4 and 7 dpi, respectively. OTA was not detected (<LOD) in mycelium from the non-permissive condition (0.93 aw GJM) at both collection points. In addition, analysis of OTA in mutant strains, lacking the *otaY* gene, after seven days of incubation in the dark on MM agar at 25 °C revealed the absence of the toxin in culture extracts. In comparison, the production of OTA was confirmed in *A. carbonarius* wild-type strain ITEM 5010 cultured under the same conditions (6467 ng/g) (Figure 4). The chromatograms reported in Figure 4 clearly show the presence of OTA in the diluted extract of the wild-type strain and the absence of OTA in the diluted extract of AC2021 mutant strain, selected as representative ∆*otaY* mutant strain. Another prominent peak, eluting at 0.85 min, was present in the chromatogram of the wild type, which is probably one of the precursors of OTA, as previously reported by Gallo et al., 2012 [15]. This peak was also present in the chromatogram of the diluted extract of the mutant strain but at a much lower concentration. Further studies are necessary to identify this compound, its possible involvement in the biosynthetic pathway of OTA, and its relationship with the *otaY* gene. In order to confirm the lack of OTA production by the mutant strain shown in Figure 4, even at very low concentrations, the fungal extract was purified and concentrated through an immunoaffinity column specific for OTA and analyzed by UPLC-FLD. No OTA was detected in the purified/concentrated extract of the mutant strain.

## 3. Discussion

Ochratoxin A is a fungal toxin known to be produced by more than twenty *Aspergillus* species of Sect. *Nigri* and *Circumdati*, and by *Penicillium nordicum, P. thymicola, P. verrucosum*. The recent availability of genome sequences from 21 OTA-producing species (19 *Aspergillus* and 2 *Penicillium*), revealed some differences in gene attributes reported by different authors. However, it established the clear involvement of five core genes (*otaA*, *otaB*, *otaC*, *otaD*, and *otaR1*) in the biosynthetic pathway of OTA [12,14,15,35]. Recently, the extensive analysis of the genomic region related to the biosynthetic cluster of OTA indicated the presence of a new gene, encoding a “SnoaL-like cyclase” domain located between the polyketide synthase (*otaA*) and non-ribosomal peptide synthase (*otaB*) genes [12]. This finding was interesting because the cyclization process of the heterocyclic structure of OTβ, in the first steps of OTA biosynthesis pathway, has not yet been clarified. Indeed, the evidence of the presence of a gene encoding a polyketide cyclase in the OTA clusters points out its possible role in the cyclization of OTA polyketide backbone. SnoaLs proteins have been characterized as belonging to a family of small polyketide cyclases, consisting of about 140 amino acids, which catalyze in *Streptomyces* cyclization steps in the biosynthesis of polyketide antibiotics [18]. The role of cyclization in polyketide biosynthesis is complex and important. However, there is limited data regarding the role of specific cyclase genes in secondary metabolite biosynthesis from filamentous fungi. More often, cyclization is thought to happen either spontaneously via intramolecular interactions within the growing polyketide or mediated by a cyclization domain that could be part of the domain structure of PKSs [36]. A second hypothesis could be the involvement of a separate gene from the *pks* gene, namely cyclase, which could be the case of the OTA biosynthesis. As example, the biosynthetic pathway for aurovertin in *Calcarisporium arbuscula* seems to be increased by a cyclase protein (*aurE*), with homology to a SnoaL-like bacterial polyketide cyclase [37,38]. While the involvement of SnoaL-like polyketide cyclases has been clearly demonstrated in the biosynthesis of polyketide antibiotics, only a few proteins with this functional domain have been reported in filamentous fungi [12,39].

The analysis of the *otaY* gene expression under permissive and non-permissive OTA conditions showed regulation of *otaY* gene congruent with the expression trend of the other OTA cluster genes, confirming an upregulation of its transcription level under OTA permissive conditions compared to non-permissive conditions. These findings supported the hypothesis that a different gene is involved in the cyclization of OTA molecule instead of *otaA* gene, responsible for polyketide biosynthesis. In this study, the role of cyclase gene *otaY* in the biosynthesis of OTA was demonstrated by using the CRISPR/Cas9 approach. The use of CRISPR/Cas9 system for the deletion of *otaY* gene was successful and revealed the usefulness of this genome editing tool for the deletion of genes located at genomic level very close to each other. Considering the genomic region where the *otaY* gene is placed, the *otaA* gene is located at about 350 bp upstream of the cyclase gene and the *otaB* gene is located at about 200 bp downstream. This short distances between important structural genes for OTA biosynthesis and the presence in this intergenic spacer of the respective UTRs regions would have made the deletion of the cyclase gene difficult with a traditional approach. The classical homologous recombination with a deletion cassette requires the fusion of 600–1000 bp of sequence flanking the target gene at both ends of a marker gene. Indeed, the CRISPR/Cas9 tool application guaranteed an almost “surgical” deletion of *otaY* gene without altering any adjacent gene coding sequences and the related gene regulation regions. Since the hygB resistance was not integrated as expected in the *otaY* deletion site, in order to dispel any doubts about any possible alteration of OTA cluster genes, we: (i) sequenced the entire *otaY* deletion site; (ii) verified the transcription of OTA cluster genes by RT-PCR; (iii) verified any significant change in expression levels of OTA cluster genes by qRT-PCR. Moreover, the integration of hygB resistance in a different site, probably due to the high microhomology of the 50 bp sequence fused to the HygB cassette with a different genomic region, led us to sequence the full genome of AC2021 strain, selected as representative mutant strain. Genome sequence analysis confirmed the occurrence of the complete deletion of *otaY* gene and the absence of any mutation in OTA cluster genes and related regulatory regions. The sequencing also confirmed that the integration of hygB resistance in a different genome region did not modify the coding sequence of the genes surrounding the integration site. To our knowledge, this is the first evidence of the involvement of a cyclase gene in the biosynthesis of OTA. The complete deletion of *otaY* gene demonstrated the inability of Δ*otaY* mutant to synthesize the OTA molecule.

It is well known that fungal polyketide synthases are multifunctional enzymes involved in the biosynthesis of fungal polyketides, harboring various functional domains with acyl transferase, ketosynthase, thioesterase, methyltransferase, and other activities.

They often use acetyl CoA as the starting substrate, malonyl CoA as the extension unit, and repeated Claisen condensation to extend the polyketide chain [40]. In general, the cyclization process of fungal polyketide metabolite is due to a Thioesterase (TE) domain in PKS multifunctional proteins [41]. In this regard, the *otaA* gene coding for a polyketide synthase, crucial for the OTA biosynthesis, lacks the TE domain, and this supports our hypothesis of the SnoaL-like cyclase involvement in the early cyclization step of OTA formation [12]. SnoaL is a polyketide cyclase that adopts a distorted alpha-beta barrel fold, and the polyketide cyclases thus form a family of enzymes with a unique catalytic strategy for aldol condensation. As detailed above, SnoaL-like cyclases are well described in the antibiotic synthesis in *Streptomyces* and other bacteria, but not in fungal secondary metabolism. Recently, a putative ester cyclase (named UvEC1) presenting a SnoaL domain has been shown to be important for the virulence of *Ustilaginoidea virens* but remarkably, its expression is inversely related to toxin production in this pathogen [39]. In conclusion, our results indicate for the first time the involvement of a cyclase gene in the biosynthesis of a polyketide mycotoxin, previously the cyclization step was always associated with the presence of a specific domain in the fungal PKS or NRPS enzymes [41]. Our results are supported by the prediction of the presence of this gene within the cluster genes in all the ochratoxigenic fungi [12] and support the original idea of an essential role of this gene in the OTA biosynthetic pathway. Our findings allowed the definition of a core cluster of six genes (*otaA*, *otaY*, *otaB*, *otaC*, *otaD*, and *otaR1*) involved in the biosynthesis of OTA. However, some questions remain that concern essentially precursor and intermediate molecules in the early stages of the biosynthetic pathway during the formation of the polyketide structure which the analyses conducted in this current work could not determine. Further investigation would permit the identification of other key pathway intermediates upstream of otβ, which is linked to phenylalanine in the reaction catalyzed by the NRPS encoded by *otaB* gene, and OTB, which is formed in the binding reaction and then converted into OTA by the action of the halogenase protein encoded by the *otaD* gene [14,15].

A logical next step will be to perform similar experiments to demonstrate the role of *otaY* gene identified in other ochratoxigenic fungi in our previous work [12]. A more in-depth knowledge can be obtained from the study of the OTA pathway in the ochratoxigenic species belonging to different sections of filamentous fungi to verify the evolutionary process responsible for the structure of OTA clusters.

## 4. Materials and Methods

### 4.1. Fungal Strains and Growth Conditions

The wild-type *A. carbonarius* ITEM 5010 from the Agro-Food Microbial Culture Collection of the Institute of Sciences of Food Production, CNR, Bari, Italy (www.ispa.cnr.it/Collection, accessed on 17 November 2021) and the mutant strain AC2021 (Δ*otaY*), generated from ITEM 5010, were used in this study. Fungal strains were grown on minimal medium (MM) agar plates [14]. The knockout Δ*otaY* strain was grown on MM supplemented with 100 mg/L hygromycin B (*hygB*).

### 4.2. Cyclase Gene Expression Analysis

The analysis of *otaY* gene expression in relation to the other OTA biosynthetic genes (*otaA*, *otaB*, *otaC*, *otaR1*, and *otaD*) was conducted under water activity conditions permissive (aw 0.99) and not permissive (aw 0.93) for OTA production. In detail, 100 μL of a conidial suspension (10^6^ conidia/mL) from wild-type strain ITEM 5010 of *A. carbonarius* were inoculated on Grape Juice Medium (GJM) prepared according to [42]. The water activity (aw) of GJM was adjusted to 0.93 by adding an appropriate amount of glucose. The aw of GJM was measured using an AquaLab 4TE aw meter at 25 °C. Inoculated plates were incubated at 18/31 °C under 10 h/14 h dark/light photoperiod. Triplicate cultures were prepared and analyzed for each experiment. Fungal mycelium was harvested after 4- and 7-day post-inoculation (dpi) and stored at −80 °C for RNA extraction and OTA analysis. Total RNA was extracted from frozen mycelium ground in liquid nitrogen using the RNeasy kit (Qiagen, Hilden, Germany) and treated with RNase-free DNase I (Qiagen) according to the manufacturer’s protocol. First-strand cDNA was synthesized using SuperScript III reverse transcriptase (Invitrogen, San Diego, CA, USA) according to the manufacturer’s protocol. The expression levels of OTA biosynthetic genes in *A. carbonarius* strains 5010 were analyzed using real-time quantitative reverse transcription-PCR (qRT-PCR); β-tubulin was used as an internal reference gene. The list of primers used in the qPCR assays is shown in Table 2. Real-time PCR was performed using the Viia 7 Real Time PCR system according to [42]. Relative gene expression analysis was performed in triplicates. The relative quantification (2^−ΔΔCT^) of gene expression was assessed by Quant-Studio™ RT-PCR Software (Thermo-Fisher Scientific, Waltham, MA, USA), considering as reference condition 4 dpi aw 0.93 at 18/31 °C under 10 h/14 h dark/light photoperiod. Analysis of variance (ANOVA) was carried out to determinate significant differences in gene expression among different growth conditions for each analyzed gene. Tukey’s test was applied to compare the mean values. Statistical significance was set at *p* ≤ 0.05. Statistical analysis was performed using the software Statistica 7.0 (StatSoft Inc., Tulsa, OK, USA).

### 4.3. Cyclase Gene Deletion: sgRNAs Selection and Fungal Transformation

The sgRNAs (Table 1) for the complete deletion of *otaY* gene (NCBI accession number MT706047 Figure S2) were designed with the Eukaryotic Pathogen CRISPR guide RNA/DNA Design Tool—EuPaGDT (grna.ctegd.uga.edu). The annotated sequence of reference strain ITEM 5010 was used to predict PAM sites and off-targets. The intergenic region between PKS and NRPS genes was selected as target region for sgRNAs prediction. In order to select optimal guide RNAs for upstream and downstream regions of *otaY* gene, any sgRNAs targeting the UTRs regions of PKS and NRPS genes and the coding sequence of *otaY* gene were excluded. The sgRNA DNA templates were PCR-assembled, transcribed, and pre-loaded on TrueCut Cas9 Protein v2 (Thermo-Fisher Scientific) as previously described by [34]. The nucleotide sequences of primer pairs IVT-cycUP-fwd/IVT-cycUP-rev and IVT-cycDW-fwd/IVT-cycDW-rev used to assemble the sgRNAs DNA templates are reported in Table 3. The two ribonucleoprotein (RNP) complexes assembled in vitro were used to transform protoplasts of *A. carbonarius* ITEM 5010 obtained according to [14]. In detail, protoplasts aliquots (200 µL, 2 × 10^6^ protoplasts) were co-transformed with 1 µg of the two RNPs (1:1) and 3μg of purified HDR-HygB repair template prepared by fusion PCR with the primer pair 5harm_cyc_Hyg_F/3harm_cyc_HygB_R (Table 3) as reported by [33]. Then, 50 µL of polyethylene glycol (PEG)-CaCl_2_ buffer (40% [*w*/*v*] PEG 4000, 50 mM CaCl_2_·H_2_O, 50 mM Tris-HCl, pH 7.5) were added, and the mixture was incubated on ice for 30 min. Afterward, 1.5 mL PEG-CaCl_2_ buffer supplemented with 1 M sorbitol was added, and the mixture was incubated at room temperature for 20 min. Subsequently, the mixture was diluted to a total volume of 3 mL with MM supplemented with 1 M sorbitol and incubated at 30 °C with gentle shaking for 1 h. After incubation, protoplasts were plated by inclusion in non-selective 1 M sorbitol MM soft agar (0.8% *w*/*v* agar) to allow regeneration of the fungal cell wall. After 16–18 h at 25 °C, a top layer of MM agar 1.2% *w*/*v* supplemented with 100 mg/L *hygB* was overlaid on transformant plates. Resistant colonies were selected after 3–6 days of incubation at 25 °C. Putative transformants were transferred to PDA containing 100 mg/L of hygromycin B. Selected mutants were subcultured as a single spore on selective MM agar supplemented with 100 mg/L of hygromycin. Monoconidial *A. carbonarius* ∆*otaY* mutant strains were cultured on selective PDA for further analysis and stored in sterile 15% glycerol at −80 °C as conidial suspensions.

### 4.4. PCR Analysis of Putative A. Carbonarius ∆otaY Mutants

Genomic DNA of wild-type ITEM 5010 and *A. carbonarius* ∆*otaY* mutants was extracted by using the Gene JET Plant Genomic DNA Purification Kit (Invitrogen, San Diego, CA, USA). The genomic region spanning the deletion site was PCR amplified with primers cyc_out_F/cyc_out_R (Table 3). PCR amplifications were carried out with Platinum SuperFi II PCR Master Mix (Invitrogen) and 500 nM of each primer. PCR amplification conditions were: 98 °C for 30 s, then 30 cycles of 98 °C for 10 s, 60 °C for 15 s and 72°C for 1 min, and a final extension at 72 °C for 5 min. Amplification products were evaluated by gel electrophoresis. Amplicons were sequenced using the BigDye Terminator v3.1 Cycle Sequencing Kit (Applied Biosystem, Waltham, MA, USA). The obtained nucleotide sequences were analyzed using CLC Genomics Workbench 7.5 (CLC, Inc., Aarhus, Denmark).

### 4.5. Gene Expression Analysis of OTA Cluster Genes in ∆otaY Mutant

An end-point Reverse Transcription (RT)-PCR and qRT-PCR were used to confirm the expression of OTA biosynthetic genes (*otaA*, *otaB*, *otaC*, *otaR1* and *otaD*) and the absence of *otaY* transcript in deletion strain.The wild-type ITEM 5010 and the selected ∆*otaY* strain AC2021 of *A. carbonarius* were grown on MM agar plates for 4 days in the dark at 25 °C. Total RNA and cDNA were obtained as described above (Chapter 4.2). PCR amplifications were performed with Platinum SuperFi II PCR Master Mix (Invitrogen) on 100 ng of cDNA and 500 nM of each primer. PCR amplification conditions were: 98 °C for 30 s, then 30 cycles of 98 °C for 10 s, 58 °C for 15 s and 72 °C for 30 s, and a final extension at 72 °C for 5 min. The primers Bt2a/Bt2b [43] were used to monitor β-tubulin gene expression as endogenous control. These primers span three introns, which also allowed checking for DNA contamination. Real-time PCR analysis was performed as reported in chapter 4.2. The relative quantification (2^−ΔΔCT^) of gene expression was assessed by Quant-Studio™ RT-PCR Software (Thermo-Fisher Scientific, Waltham, MA, USA), considering as reference condition the expression level of each gene in wild-type strain ITEM 5010.

### 4.6. Genome Sequencing and Assembly

The genome of AC2021 *A. carbonarius* ∆*otaY* mutant strain was sequenced by Illumina HiSeq 2 × 150 paired end reads. After quality filtering, the reads were de-novo assembled by using the de Bruijn graph-based algorithm implemented in MaSuRCA Assembler—v3.2.9 [44].

Obtained scaffolds were used to map the excision site of *otaY* gene and the integration site of hygromycin B gene marker. Genes surrounding *otaY* excision and *hygB* integration sites were manually annotated to check if the editing procedure and induced DNA repairing events modified any other locus of OTA cluster and/or known genes.

### 4.7. OTA Analysis

The production of OTA by strain ITEM 5010 was monitored on 0.99 and 0.93 aw GJM after 4- and 7-day incubation at 18/31 °C under 10 h/14 h dark/light photoperiod.

The method of Gallo et al., 2014 [35], for the determination of OTA in mycelium was slightly modified and used herein for OTA determination. The samples were analyzed directly by UPLC-FLD system without immunoaffinity cleanup. In particular, 500 mg of frozen mycelium grounded in liquid nitrogen and stored at −80 °C (the same used for gene expression analysis) were transferred to a 12 mL test tube. About 0.5 g of mycelium were extracted with 10 mL of mixture MeOH:ACN:H_2_O (30:30:40, *v*/*v*/*v*) by shaking for 120 min at room temperature and centrifuged for 15 min at 3000× *g*. An aliquot of 600 µL of supernatant was diluted with 400 µL of mixture ACN:H_2_O:Acetic Acid (35:62.5:2.5, *v*/*v*/*v*) and 10 µL, corresponding about 0.00023 g of solid sample, were injected into Waters Acquity UPLC system (Milford, MA, USA) full loop injection system. The chromatographic column was an Acquity UPLC BEH RP-18 (100 mm × 2.1 mm i.d., 1.7 μm column, Acquity) with an Acquity UPLC column in-line filter (0.2 μm).

Moreover, OTA production was verified for wild-type strain ITEM 5010 and four independent Δ*otaY* strains (AC2021-AC2024) grown on MM agar plates in the dark at 25 °C, performing the cleanup step reported in Official Method AOAC 2001.01 [45]. After 7 days of growth, 5 agar plugs with mycelium (about 500 mg) were collected from each replicate and extracted as reported above. An aliquot of 6 mL of sample extracts in MeOH:ACN:H_2_O (30:30:30, *v*/*v*/*v*) were diluted with 44 mL of a water solution containing PEG (1%) and NaHCO_3_ (5%), mixed, filtered through a glass microfiber filters GF/A and 10 mL of diluted sample cleaned-up by OchraTest immunoaffinity columns (Vicam, Watertown, MA, USA). The column was washed with 10 mL of an aqueous solution containing NaCl (2.5%) and NaHCO_3_ (0.5%), followed by 10 mL of ultrapure water at a flow rate of 1−2 drops/s. The eluates were discarded, and the OTA was recovered in a vial by passing 2 mL of methanol through the column. The eluate was collected in a glass vial, evaporated to dryness at 50 °C under a gentle stream of nitrogen, and redissolved in 500 µL of a UPLC mobile phase (H_2_O:ACN:Acetic Acid, 99:99:2, *v*/*v*/*v*). The purified extracts were filtered through a 0.22 µm pore filter. Ten microliters, corresponding to about 0.001 g of mycelium, were injected into the UPLC-FLD.

## Figures and Tables

**Table 1:** sgRNAs used in this study.

ID	Protospacer Sequence 5′→3′	PAM Site
sgRNAotaY_up	ATTAGCCCTACACGTCAACC	GGG
sgRNAotaY_dw	GTTAGTTGGGATTCGCTGCT	GGG

**Table 2:** Primers for qRT-PCR and RT-PCR used in this study.

Primer	Sequence (5′–3′)	Concentration	Target Gene	Reference
RT_OTApks_Ac_FOR	CGTGTCCGATACTGTCTGTGA	200 nM	*otaA*	[35]
RT_OTApks_Ac_REV	GCATGGAGTCCTCAAGAACC	200 nM		
RT_Ac_otaY_for	ACCATCCTCACCACCCTTGT	200 nM	*otaY*	[14]
RT_Ac_otaY_rev	GGGACTCTGGGCTAACACCT	200 nM		
RT_nrps_Ac_FOR	ACGGGTCGCTGCTCTATATC	200 nM	*otaB*	[14]
RT_nrps_Ac_REV	ACTCACCACATCAACCACGA	200 nM		
RT_AcOTAp450_F	GTGGTTATCCCGCCCAATAC	200 nM	*otaC*	[14]
RT_AcOTAp450_R	TGCCAGATTCATCCCGATAC	200 nM		
RT_Ac_OTAbZIP_for	AATGGAACCAGCATTGATCTC	250 nM	*oraR1*	[14]
RT_Ac_OTAbZIP_rev	GACCCAAGCATTCGCTCTA	250 nM		
RT_hal_Ac_FOR	GAACGCCAGTAGAGGGACAG	200 nM	*otaD*	[14]
RT_hal_Ac_REV	ATGGAGGTGGTGTTGTTGTG	200 nM		
RT3 BT Ac_F	CAAACCGGCCAGTGTGGTA	200 nM	*BenA*	[14]
RT3 BT Ac_R	CGGAGGTGCCATTGTAAACA	200 nM		

**Table 3:** Primers used in this study. Primer sequences specific for amplification of the *hygB* cassette are in bold.

Primer ID	Nucleotide Sequence 5′→3′
IVT-cycUP-fwd	TAATACGACTCACTATAGATTAGCCCTACACGTC
IVT-cycUP-rev	TTCTAGCTCTAAAACGGTTGACGTGTAGGGCTAA
IVT-cycDW-fwd	TAATACGACTCACTATAGGTTAGTTGGGATTCGC
IVT-cycDW-rev	TTCTAGCTCTAAAACAGCAGCGAATCCCAACTAA
5harm_cyc_Hyg_F	CTCACCAGGCTGTGGCAAGCAGTTGGCGTGTATATTAGCCCTACACGTCA**ACAGTTTAGCTTGCCTCGTC**
3harm_cyc_HygB_R	TACTTCATATATCCAACCAAACAAAACACATACCCTAGTTTCTCACCCAG**TCCAGTATAGCGACCAGCATT**
cyc_out_F	CATTGTGCTGGACTTTGGGC
cyc_out_R	CCGGCTTTACTTTCATGGCG

## Data Availability

The sequence data are available at NCBI SRA under BioProject ID: PRJNA738506.

## Supplementary Materials

The following are available online at https://www.mdpi.com/article/10.3390/toxins13120892/s1, Figure S1: Colonies (clockwise from the top) on Czapek yeast extract agar (CYA), malt extract agar (MEA), and yeast extract sucrose (YES) after three and five days of incubation at 25°C in the dark of *A. carbonarius* wild type strain ITEM 5010 (A and C) and *A. carbonarius* ∆*otaY* deletion mutant strain AC2021 (B and D), Figure S2: Nucleotide sequence of *Aspergillus carbonarius otaY* polyketide cyclase (NCBI accession number MT706047).
