# Supplementary material for: Evidence of the Involvement of a Cyclase Gene in the Biosynthesis of Ochratoxin A in Aspergillus carbonarius
Source: Toxins (Basel). 2021 Dec 13;13(12):892. doi: 10.3390/toxins13120892 (PMC8705981; doi:10.3390/toxins13120892)
Supplement: Supplementary file 1 [file toxins-13-00892-s001.zip › toxins-1491377-supplementary.pdf]

toxins

MDPI

Supplementary Materials: Evidence of the Involvement of a Cyclase Gene in the Biosynthesis of Ochratoxin A in *Aspergillus carbonarius*

Massimo Ferrara, Antonia Gallo, Carla Cervini, Lucia Gambacorta, Michele Solfrizzo, Scott E. Baker and Giancarlo Perrone

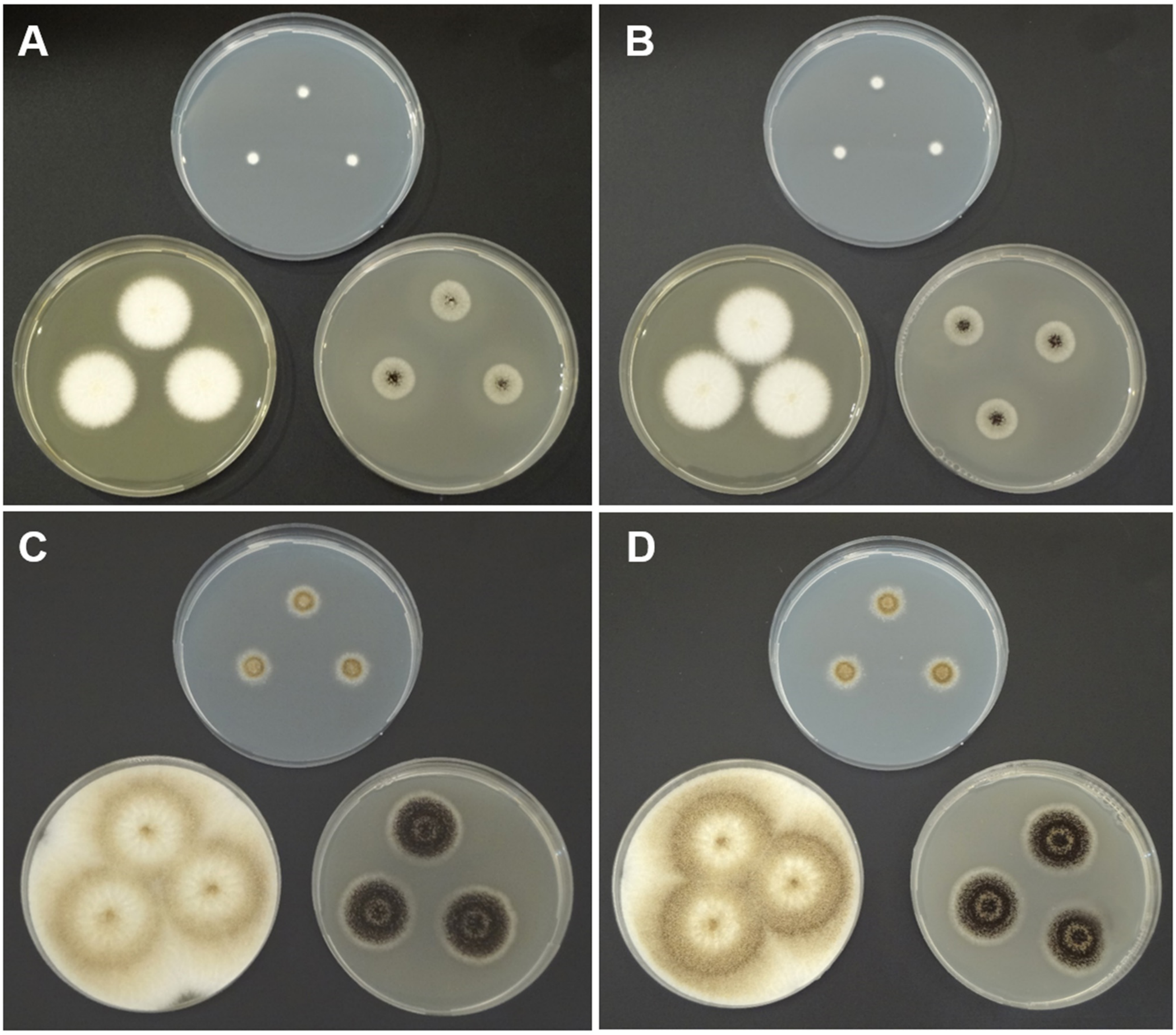

Figure S1. Colonies (clockwise from the top) on Czapek yeast extract agar (CYA), malt extract agar (MEA), and yeast extract sucrose (YES) after three and five days of incubation at 25°C in the dark of *A. carbonarius* wild type strain ITEM 5010 (A and C) and *A. carbonarius* Δ*otaY* deletion mutant strain AC2021 (B and D).

```
1    ATGACCACACCCACACCCACAACCCTCAAACACCACGCCACCACCATCCTCACCACCCTTGTCAACGACC   70
71   GCCAAACCGCCGCCATCGAACACCTCCTCCACCCCACCATCACCCTCAAACACAATGACCTCCCCGCCAT  140
141  GAGTAAATCCGAGCTCATTGCCTTCTGGCCCGAGGTGTTAGCCCAGAGTCCCCATTTCCGGGTCCAGATC  210
211  CGGGATGTTATTGCCGAGGGGAATAAGGTGTGGGTTTATTCGCGCGTGGAGGGGAGACTTGGGGAGGGGG  280
281  TGATGGATGATTTTCATATGATGGTTTTTGATGAGGAGGGGTTGGTGGTGAGGAGTACGGGGGTGCAGAG  350
351  GGTTGTTGAGGGGGAGATGTAG                                                  372
```

Figure S2. Nucleotide sequence of *Aspergillus carbonarius otaY* polyketide cyclase (NCBI accession number MT706047).
